# Mast cell stabilizers: from pathogenic roles to targeting therapies

**DOI:** 10.3389/fimmu.2024.1418897

**Published:** 2024-08-01

**Authors:** Mengda Cao, Yao Gao

**Affiliations:** ^1^ Department of Pharmacy, Zhongda Hospital, School of Medicine, Southeast University, Nanjing, China; ^2^ Department of Endocrinology, Children’s Hospital of Nanjing Medical University, Nanjing, China

**Keywords:** MC stabilizers, activation, mechanism, targeted, clinical application

## Abstract

Mast cells (MCs) are bone-marrow-derived haematopoietic cells that are widely distributed in human tissues. When activated, they will release tryptase, histamine and other mediators that play major roles in a diverse array of diseases/disorders, including allergies, inflammation, cardiovascular diseases, autoimmune diseases, cancers and even death. The multiple pathological effects of MCs have made their stabilizers a research hotspot for the treatment of related diseases. To date, the clinically available MC stabilizers are limited. Considering the rapidly increasing incidence rate and widespread prevalence of MC-related diseases, a comprehensive reference is needed for the clinicians or researchers to identify and choose efficacious MC stabilizers. This review analyzes the mechanism of MC activation, and summarizes the progress made so far in the development of MC stabilizers. MC stabilizers are classified by the action mechanism here, including acting on cell surface receptors, disturbing signal transduction pathways and interfering exocytosis systems. Particular emphasis is placed on the clinical applications and the future development direction of MC stabilizers.

## 1 Introduction

Mast cells (MCs) are bone-marrow-derived haematopoietic cells involved in a multitude of diseases/disorders, including allergies, inflammation, migraine headache, cardiovascular diseases, autoimmune disease, cancer and even death (**Table 1**) (9, 18). They are widely distributed in tissues, especially at sites exposed to the external environment, such as the skin, digestive tract and respiratory tract (19). As innate immune cells, MCs are involved in the early and rapid sensing of external invaders such as bacteria, viruses, fungi, parasites and other allergic proteins (20).

**Table 1:** Common symptoms and diseases caused by MC mediators.

System	Mediators	Symptoms and diseases	References
**Systemic**	Histamine, Proteases, ILs, Cytokines	Anaphylaxis, Systemic mastocytosis, Tissue fibrosis	(1–3)
**Cardiovascular**	Tryptase, Histamine, TGF-β, Matrix metalloproteinases, Renin	Atherosclerosis, Supraventricular tachycardia and cardiac arrest, Irregularities of blood pressure regulation, Chronic heart failure, Acute coronary syndromes, enhancing angiogenesis (stimulating tumor proliferation)	(4–8)
**Neurologic:**	Adipocytokines, Histamine, proteases	Migraine headache, Brain fog, Paresthesias, Peripheral neuropathy, Brain inflammation and autism	(9–12)
**Dermatologic**	Histamin, TNF-α, Cytokines, Chemokines	Angioedema, Dermatographism, Flushing, Pruritus, Urticaria	(11, 13)
**Gastrointestinal**	Histamine, IL-16, Eosinophilic chemotactic factor	Abdominal pain, Bloating, Diarrhea, Esophagitis, Nausea, Vomiting, Crohn’s Disease	(10, 14)
**Musculoskeletal**	TNF-α, VEGF, TGF-β, IL-6, MMP9, Chymase	Bone/muscle pain, Degenerative disc disease, Osteoporosis	(15, 16)
**Respiratory**	PGD2, Histamine, Proteases, ILs, Cytokines, LTs	Hoarseness, Sore throat, Throat swelling, Wheezing, Allergic asthma, Allergic rhinitis	(17)

Upon activation, MCs release biologically active compounds, and exert physiological and pathological functions. The mediators of MCs can be classified into three types: i) preformed mediators stored in secretory granules; ii) neoformed or lipid mediators derived from membrane lipids; and iii) neosynthesized mediators produced following transcriptional activation. Histamine, chymase and tryptase are well-known MC preformed mediators. Histamine can induce vasodilation, bronchoconstriction, smooth muscle contraction and augment mucus secretion (1, 2), all of which are commonly associated with allergic and inflammatory reactions. In addition, histamine can also stimulate tumor proliferation by enhancing angiogenesis (4, 5). Chymases and tryptases are serine proteinases and exclusively expressed by MCs, which can produce the coronary constrictor angiotensin and induce proteolytic changes in high density lipoprotein particles (6, 7). They are related to a number of pathological states including inflammation, arthritis, innate immune defence, glomerulonephritis, abdominal aortic aneurism formation and tumor angiogenesis (21, 22). Based on serine proteinase composition, MCs can be divided into two subsets: a subset that contains the tryptase and chymase (MCTC), and a subset that contains only tryptase (MCT) (23). Histamine and tryptases released by activated meningeal (dural) MCs play important roles in the migraine headache pathophysiology mainly through the complex bidirectional relationship with calcitonin gene-related peptide (CGRP) (9). The activation of mast cell signaling pathways leads to the rapid production and release of neoformed mediators, representing by prostaglandins (PGs) and leukotrienes (LTs) (10). PGs contribute to mucus production, leukocyte recruitment, increased vascular permeability and nerve cell activation (10). LTs exert local effects on the vascular endothelium by promoting the recruitment of eosinophils and neutrophils, which is beneficial to host defend against bacterial infections (10). Neosynthesized mediators produced following transcriptional activation and regulated by the type of stimuli and receptor including cytokines, CXC-chemokine ligands (CXCL) and CC-chemokine ligands (CCL) (11). Transforming growth factor-β (TGF-β) released by MCs has been shown to promote Treg function contributing to controlling autoimmune and allergic inflammation (24). More detrimentally, it is extensively implicated in the pathogenesis of fibrosis (3). Other cytokines, associated with type 1and 2 T-helper cell responses such as interferon-gamma (IFN-γ), IL-2, IL-4, IL-3 and TNF-α, are primarily involved in inflammatory responses that include alopecia areata, obesity, diabetes and laminitis (25). The chemokines CCL5 and CXCL8 can recruit immune cells to sites of infection (26).

The diverse cellular functions and ubiquitous distribution make MCs a hotspot for the treatment of numerous diseases, especially allergic diseases (24). To date, the clinically available MC stabilizers are limited. Due to the rapidly increasing incidence rate and widespread prevalence of MC-related diseases, a comprehensive reference is needed for the clinicians or researchers to identify new effective MC stabilizers. The present review classifies MC stabilizers based on their mechanism. Particular emphasis is placed on the clinical applications and the future development direction of MC stabilizers.

## 2 Process of MC activation

**Figure 1:**
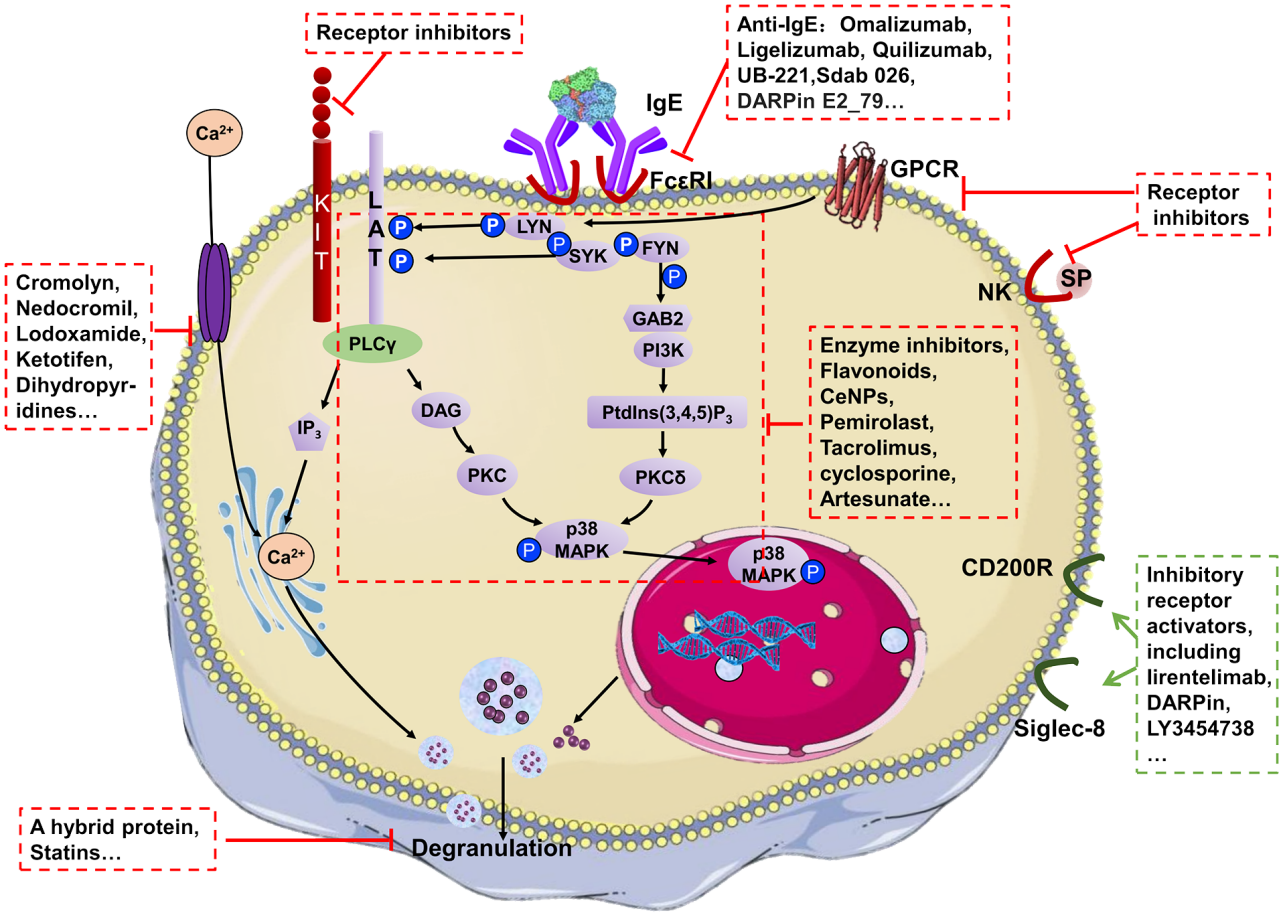
A highly simplified mechanism for MC degranulation and the action mechanism of MC stabilizers.

MC activation is regulated by surface receptors (27), including FcϵRI, KIT, Mas-related G protein coupled receptor X2 (MRGPRX2) and natural killer (NK) receptor. The FcϵRI-dependent pathway is the most recognized MC activation pathway with the crosslink of FcϵRI caused by the recognition of allergens to bound IgE. The crosslink leads to spleen tyrosine kinase (Syk)-dependent phosphorylation and activation of the SRC-family kinases FYN and LYN, causing the phosphorylation of adaptor proteins, including linker For Activation of T-Cells (LAT) and Grb2-related adaptor protein (GAB2) (28). The downstream pathways consist of the PLCγ and the phosphatidylinositol 3-kinase (PI3K) signalling pathways (28). Signal molecule recruitment is followed by activation of second messenger molecules, including inositol triphosphate (IP_3_), diacylglycerol (DAG) and PtdIns, which activate protein kinase C (PKC) and increase intracellular Ca^2+^. Along with Ca^2+^ mobilization, the degranulation machinery is triggered. Granules containing mediators move to the plasma membrane in a microtubule-dependent manner along with the production of lipid mediators and cytokines (**Figure 1**).

FcϵRI -independent pathways mediated by KIT, NK or MRGPRX2 have also been found to serve pivotal roles in the pathophysiology of various allergic and inflammatory conditions (**Figure 1**) (29). KIT, NK and MRGPRX2 can also induce autophosphorylation at multiple tyrosine residues in the cytoplasmic tail resulting in the recruitment of signal molecules and leading to MC degranulation (**Figure 1**). These receptors can both be the ‘prime’ method for MC activation and complementary of FcϵRI-dependent pathways. Among them, MRGPRX2 is a prominent receptor responsible for FcϵRI-independent allergic reactions, including itch, rosacea, urticaria and adverse drug reactions (30). It can be activated by a wide range of stimuli including cysteine proteases, neuropeptides, small cationic molecules and peptides with amphipathic properties, and drugs, playing important roles in host defence, immunomodulation, inflammatory diseases and pseudo-allergic drug reactions (31, 32). The pathway of MRGPRX2-mediated activation is similar to that of the FcϵRI-dependent pathway, including Ca^2+^ mobilizing and activation of downstream signals such as Erk1/2, JNK, p38 and PI3K/AKT (33).

## 3 MC-stabilizing agents

Regarding various MC activation pathways, a diverse array of MC stabilizers has been reported. The mechanism of action for MC stabilizers can be classified into three categories: i) Blocking external stimulus signals into cells; ii) inhibiting intracellular signalling pathways; and iii) disturbing degranulation (**Figure 1**, **Table 2**).

**Table 2:** Current therapeutic strategies targeting MCrefer.

	Compounds	Type	Mechanism of action	Characteristics	References
*Blocking external signals into the MC*					
Anti- IgE	Omalizumab	mAb	Binding to IgE	Reducing free IgE	(34)
	Ligelizumab	mAb	Binding to IgE	88-fold higher affinity for human IgE than that of omalizumab	(35, 36)
	Quilizumab	mAb	Binding to IgE Cytotoxicity on mIgE-expressing B cells	>100-fold higher affinity for human IgE than that of omalizumab Suppressing IgE production	(37)
	UB-221	mAb	Binding to IgE Binding to CD23 on B cell	Reducing both free and bound IgE, Downregulate CD23-meidated IgE synthesis	(34, 38, 39)
	sdab 026	Single-domain antibody, Recombinant proteins	Binding to IgE overlap with the CD23 binding site	Reducing both free and bound IgE,	(40)
	DARPin E2_79 bi53_79	Recombinant proteins	Binding to IgE	Reducing both free and bound IgE,	(41, 42)
	C3a7 and C3a9	C3a-derived peptides	Interacting with the β chain of FcϵRI	Lack of studies	(43–45)
Inhibition of Ca^2+^ influx	Cromolyn	Small molecular	Preventing Ca^2+^ influx and activating GPR35	More potent effect on lung MC	(46–56)
	Nedocromil	Small molecular	Preventing Ca^2+^ influx and activating GPR35	Tachyphylaxis in lung and tonsillar MCs, but not in adenoidal and intestinal MCs	(57–60)
	Lodoxamide	Small molecular	Preventing Ca^2+^ influx	Dual stabilizing action on both MCs and eosinophils, allergic conjunctivitis	(61, 62)
	Ketotifen	Small molecular	Preventing Ca^2+^ influx	MC stabilizing properties and strong H1 receptor antagonism	(63–69)
	Dihydropyridines	Small molecular	L-type Ca^2+^ channel blockers	Not the first choice for MC targeting therapy	(70, 71)
MRGPRX2 antagonists	Isoliquiritigenin, shikonin, imperatorin, roxithromysin	Small molecular	Binding to MRGPRX2	low affinity and low selectivity; can interact with a diverse group of ligands	(30, 33, 72)
KIT antagonists	Pyrimidine derivatives, Quinazoline derivatives, Indolinone derivatives, Indole derivatives, Quinoline derivatives	Small molecular	Binding to KIT	Known as anti-cancer agents, More potent in chronic allergic asthma and mastocytosis; Virtual eradication of tissue MCs and a sustained decrease in serum tryptase levels,	(73–78)
NK-1 receptor antagonists	CP99994	Small molecular	Blocking the NK-1 receptor	Stress induced inflammatory skin disease	(79, 80)
Inhibitory receptors	LY3454738(anti-CD200R) Lirentelimab(anti-Siglec-8)	mAb	ITIM tyrosine phosphorylation	Cross-link activated receptors with inhibitory receptors can abrogate activating signal	(81–87)
*Interfering intracellular signaling pathways*					
SFK inhibitors	Ibrutinib, acalabrutinib, etc. and sophorae flos	Small molecular or natural compounds	Inhibiting activities of Lyn, Fyn, BTK or Hck,	Broadly prevent IgE-mediated MC degranulation, such as acalabrutinib	(88–93)
Syk inhibitors	2,4-diaminopyrimidine (R112, R406, R788,R343), piceatannol and curcumin	Small molecular or natural compounds	Competing with ATP-binding sites of syk	Allergic rhinitis,autoimmune diseases, inflammatory diseases, cancer, and infectious diseases	(94–104)
PKC family inhibitors	Bisindolylmaleimide, calphostin C, sphingosine, quercetin and myricitrin	Small molecular or natural compounds	Inhibiting catalytic domain or regulatory domain of PKC	More potent effect on skin and lung MCs	(105–112)
PI3K inhibitors	Idelalisib	Small molecular	Binding to PI3K p110d in its ATP-binding site	Allergic rhinitis	(113–118)
JA3K,CERK, PDE5 inhibitor	WHI-131, K1, vardenafil	Small molecular	Inhibiting JA3K, CERK, PDE5 separately	Lack of studies	(119–122)
Nanoenzyme	CeNP	Nano particle	phosphatase-mimetic activity.	Low cost, high stability, easy to preparation and modification	(123, 124)
*Disturbing MC degranulation*					
Hybrid proteins	Fusion of mast-cell targeted proteins and proteases	Recombinant protein	Targeting MC and interfering its exocytosis	Lack of studies	(125)
Statins	Fluvastatin, simvastatin and atorvastatin.	Small molecular	Interfering with microtubule formation.	Treating hypercholesterolemia, not the first choice for MC targeting therapy	(126–131)
*Mixed action mechanism*					
Flavonoids	fisetin, genistein, luteolin, quercetin and kaempherol	Natural compounds	Interfering cell-to-cell interaction between MC and T cell membranes, inhibiting the activity of NF-κB and MAPKs	Effective in the treatment of allergic conjunctivitis, rhinitis, otitis, asthma and food allergy.	(132–142)
Pemirolast	[1,2-a]pyrimidin-4-one derivative	Small molecular	Inhibiting Ca^2+^ mobilization, arachidonic acid release and metabolism; suppressing phosphodiesterase activity; increasing cAMP levels	Allergic asthma and ragweed allergic conjunctivitis More suitable for continuous therapy	(143–145)
Others	Tacrolimus and cyclosporine, Artesunate		Not the first choice for MC targeting therapy		(146–149)

### 3.1 Blocking external signals into the MCs

#### 3.1.1 Anti- IgE

IgE plays a central role in MC activation. The ability to reduce circulating IgE with a humanized monoclonal antibody (mAb) such as omalizumab, ligelizumab, quilizumab and UB-221 represents a new approach for stabilizing MCs (150). Omalizumab is a first generation anti-IgE mAb that was originally designed to reduce patients’ sensitivity to inhaled or ingested allergens (34). It selectively binds to the Cϵ3 domain of soluble IgE, thus immobilizing and preventing IgE-FcϵRI binding (151). Combining omalizumab (300 mg/month subcutaneously) with anti-inflammatory agents and/or pimecrolimus, a calmodulin inhibitor, can achieve better therapeutic efficacy in MC-associated diseases (152), including allergic asthma, allergic rhinitis and urticaria. Apart from allergic diseases, omalizumab also plays an important role in infectious diseases, such as aspergillosis. Omalizumab treatment reduced exacerbations and oral corticosteroid use, improved lung function and asthma control in patients with allergic bronchopulmonary aspergillosis (ABPA) and was well-tolerated (153). It is also demonstrated to enhance plasmacytoid dendritic cell antiviral responses (154).

Ligelizumab is a second-generation anti-IgE mAb with an 88-fold higher affinity for human IgE than omalizumab (35, 36). In a phase 2b clinical trial (NCT02477332), ligelizumab therapy of 72 mg or 240 mg had a higher percentage of complete control of symptoms in patients with chronic spontaneous urticaria compared to omalizumab at a dose of 300 mg, or placebo, administered subcutaneously every 4 weeks (155). Different from omalizumab, ligelizumab cannot dissociate the combined IgE from FcϵRI. Quilizumab is also classified as a second-generation anti-IgE mAb. It selectively binds to the Cϵ3 and Cϵ4 domains of human IgE with a higher affinity (>100-fold) than omalizumab (37). Quilizumab can suppress IgE production by its antibody-dependent cell mediated cytotoxicity on IgE-expressing B cells. However, the results of clinical trials (NCT01987947, NCT01582503) showed that its effect on IgE production and B cells could not bring a clinically meaningful benefit for adults with refractory chronic spontaneous urticaria (CSU) and allergic asthma when used at a dose of 300 mg monthly (156, 157). UB-221 is a third-generation anti-IgE mAb (38). Compared with the previous two generations, UB-221 binds to IgE with a higher affinity and reduces faster the IgE levels in circulation (34). UB-221 binds to CD23 on B cells and downregulates CD23-mediated IgE synthesis (39). Phase 1 studies are investigating the characteristics and effect of intravenous UB-221 in patients with CSU (150). Treatment with IgE mAb provides a new era for the management of severe allergic conditions (158).

Apart from traditional mAbs, recombinant proteins have been reported to possess anti-IgE activity, including recombinant humanized single-domain antibody (sdab) and designed ankyrin repeat proteins (DARPins) (159). The sdab 026 was reported to reduce both free and bound IgE, which is more efficient than omalizumab (40). Sdab 026 targets IgE by binding to an epitope within the Fc domains that markedly overlap with the CD23-binding site instead of the FcϵRI-binding site (40). The DARPin E2_79 is a fusion of two anti-IgE DARPins that not only prevents binding of free IgE to FcϵRI, but also removes receptor-bound IgE from the cells in a concentration- and time-dependent manner (41). Moreover, the fusion of DARPin E2_79 with the non-inhibitory anchor DARPin E3_53 results in a bi-paratopic anti-IgE binder, bi53_79, with markedly enhanced disruptive efficacy (42). Compared with mAbs, these recombinant proteins exhibit increased stability and high production yield in simple expression systems. Advanced strategies for multiple targeting and half-life extension can be easily applied to them. Delivery in functional form via mucosal and airway tissues may be possible using these small-size IgE inhibitors promoting anti-IgE application.

A patent reported that the C3a-derived peptides C3a7 and C3a9 can interact with the β-chain of FcϵRI on MCs and decrease the probability of IgE FcϵRI binding, resulting in suppression of signal transduction (43–45). Although C3a7 and C3a9 can inhibit MCs’ function, in-depth studies are lacking and their specific functions are largely unknown.

#### 3.1.2 Prevention of Ca^2+^ influx into MCs

The increase in intracellular Ca^2+^ is a key step for MC activation. Intracellular Ca^2+^ is necessary for microtubule assembly, microfilament contraction, vesicle fusion to the cell membrane and subsequent degranulation (160). Prevention of Ca^2+^ influx into MCs is the action of mode of most clinically approved MC stabilizers.

##### 3.1.2.1 Cromolyn

**Figure 2:**
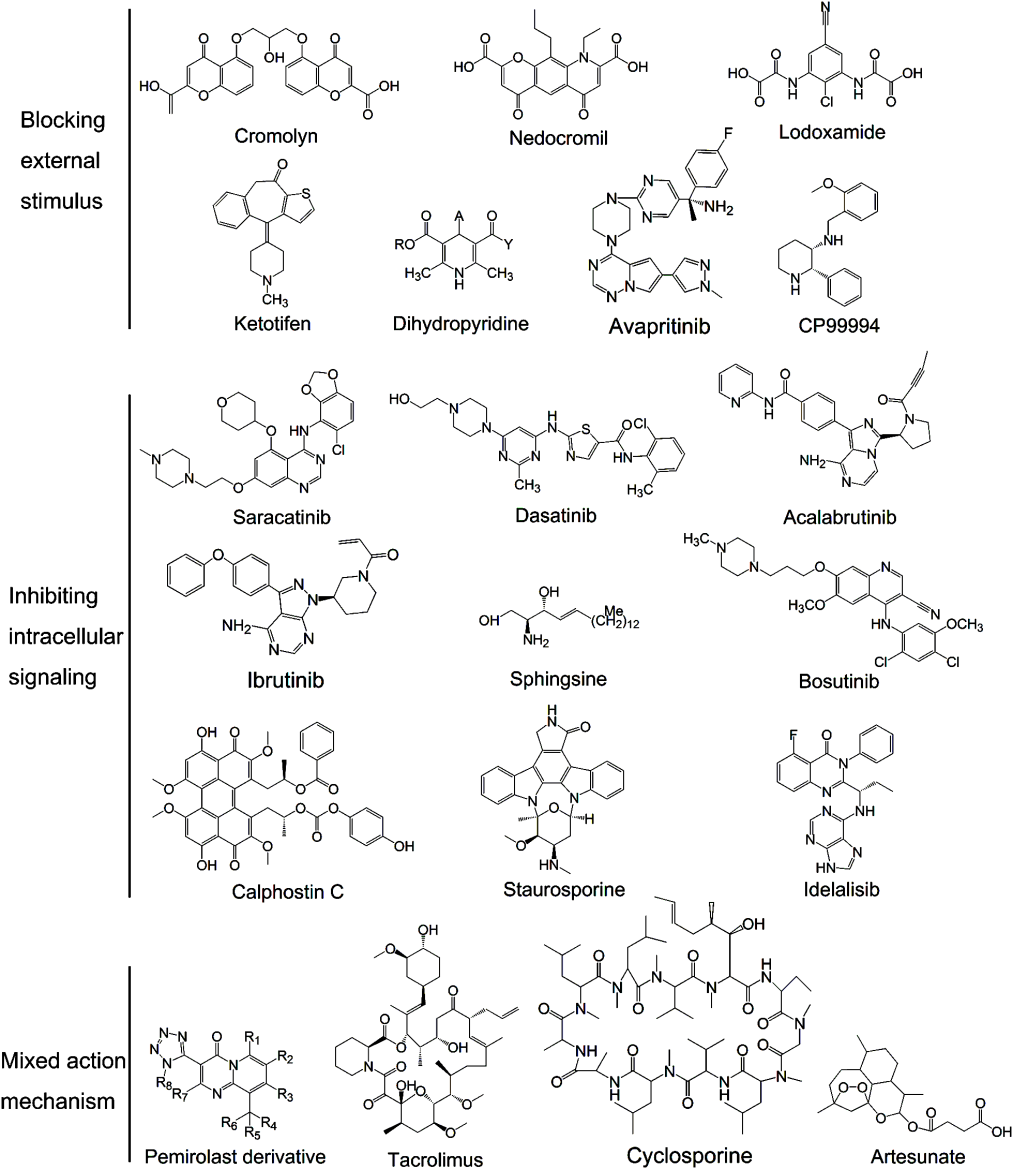
The structure of some MC stabilizers.

In 1965, cromolyn, also known as 5,5 - [(2 - hydroxytrimethylene) bis - (oxy)]4 - oxo - 4H-1-benzopyran-2-carbox-ylic acid (**Figure 2**), was first synthesized during experiments for the drug khellin used for cardiovascular disease treatment. It is the most commonly used MC stabilizer that binds specifically to the Ca^2+^-binding protein on MC membranes, forming a ternary complex with Ca^2+^. The ternary complex creates a blockage that stabilizes MC membranes and prevents degranulation (46). Recently, it was also shown to have agonist activity at GPR35 (47) which is predominantly expressed in the gastrointestinal tract and is closely related to inflammatory bowel diseases (48). GPR35 is coupled with numerous effectors after agonist stimulation, causing a series of downstream events, including ion channel inhibition and transient Ca^2+^ reduction (49). However, this discovery differs from previous findings showing that GPRs are activated receptors and GPR35 is upregulated upon stimulation with allergens (50). Therefore, further investigation concerning this potential mechanism is necessary.

Cromolyn has a potent MC stabilizing effect, especially on lung MCs. Thus, it is widely used for the treatment of allergic diseases such as rhinitis and asthma. It can improve acute and chronic injury in lung transplant animal models, but the precise mechanism of action remains unknown (51). Due to its large molecular weight, highly hydrophilic and ionisable character, cromolyn is poorly absorbed by the gastrointestinal tract. Thus, inhalation is the preferred delivery method. Cromolyn must be taken 4-8 times daily due to its short half-life (52) which results in a poor compliance. Recent studies (53) found that cromolyn was able to form niosomes, giving this drug a higher percutaneous permeation profile and the possibility for passive transdermal delivery. Besides, a series of metal complexes derived from cromolyn were recently designed, including cromolyn-Zn, -Mg and -Ca. These new metal complexes can prolong the action time and reduce adverse reactions (54). Compared with other MC stabilizers, cromolyn is preferred for patients with cardiovascular diseases (55), obesity and diabetes (56).

##### 3.1.2.2 Nedocromil

Nedocromil sodium, also known as [9-ethyl-6, 9-dihydro-4, 6-dioxo-10-propyl-4H- pyrano (3,2-g) quinoline-2,8- dicarboxylic acid; **Figure 2**], is classified as a benzopyrone and is a second-generation cromolyn drug. Its mechanism of action is similar to cromolyn in preventing Ca^2+^ influx and activating GPR35. Apart from Ca^2+^, nedocromil is capable of inhibiting chloride ion flux in MCs (57). Electrolytes with low ion numbers, such as K^+^ and Cl^-^, are likely to affect cell membrane potential and inhibit Ca^2+^ influx indirectly.

Nedocromil shows tachyphylaxis in lung and tonsillar MCs, but not in adenoidal and intestinal MCs (58). It is more powerful than cromolyn sodium in inhibiting inflammatory mediator release in bronchial mucosa with a better safety profile (59). Thus, nedocromil can be used as an effective therapeutic for tonsillitis, asthma and asthmatic bronchitis. Moreover, a patent stated that nedocromil sodium was the first choice MC stabilizer for animal laminitis (60). However, detailed experiments were not provided, and the application requires more evidence.

##### 3.1.2.3 Lodoxamide

Lodoxamide or 2-[2-Chloro-5-cyano-3-(oxaloamino)anilino]-2- oxoacetic acid (**Figure 2**), is another clinically effective MC stabilizer. Its derivatives, lodoxamide ethyl and lodoxamide tromethamine, have high anti-allergic activity. They can inhibit Ca^2+^ influx into MCs in response to antigen and activating GPR35 (48, 161). Lodoxamide can be used to control various allergic responses due to its dual stabilizing action on both MCs and eosinophils. Especially, it is widely used for allergic conjunctivitis with a better effect than cromolyn and nedocromil (61). In addition, animal studies (62) showed that injection of lodoxamide inhibited CP48/80-induced hypotension, which indicated the therapeutic effect of lodoxamide on cardiovascular disease.

##### 3.1.2.4 Ketotifen

Ketotifen or [4-(1-methyl-4-piperidylidene)-4h-benzo[4,5]cyclohepta [1,2-b]thiophen-10(9H)-one fumarate; **Figure 2**], is a benzocycloheptathiophene derivative with MC stabilizing properties and strong Histamine type 1 (H1) receptor antagonism (63). It blocks Ca^2+^ channels essential for MC degranulation resembling the function of cromolyn, which is its main mechanism of action (64). Besides, ketotifen was also reported to suppress the process of exocytosis in a dose-dependent manner by counteracting the plasma membrane deformation in degranulating MCs (65). Ketotifen is widely used in IgE-mediated allergic reactions, including dermatitis, urticaria, asthma, and food or drug allergy (66). Its ability to inhibit passively induced skin allergy and allergic airway obstruction is 6 and 50 times stronger than that of cromolyn, respectively. Apart from the anti-allergy effects, it also plays major roles in prevention of UV-induced wrinkle formation, reduction of joint capsule fibrosis and improvement of sperm quality, chromatin integrity and pregnancy rate after varicocelectomy (67–69). Due to its central nervous system inhibitory effects and anticholinergic effects, ketotifen should not be taken prior to driving, operating heavy machinery or performing athletic endeavours.

##### 3.1.2.5 Dihydropyridines

Dihydropyridines (**Figure 2**) are L-type Ca^2+^ channel (LTCC) blockers usually used in the treatment of hypertension. MCs express dihydropyridine-sensitive LTCCs, which is likely the mechanism by which dihydropyridines stabilize MCs (70). In animal experiments, dihydropyridines showed therapeutic effects in the treatment of bronchoconstriction and ocular allergies (71). However, these drugs are not the first choice in the treatment of MC-related diseases and further clinical studies are needed.

#### 3.1.3 Promoting the potassium efflux

Apart from Ca^2+^ channels, the K^+^ and Cl ^−^ channels may play a key role in activation processes of MCs since these channels modulate cell membrane potential and inhibit Ca^2+^ influx indirectly. A recent study showed that a selective TWIK-related spinal cord potassium (TRESK) channel activator, cloxyquin, dose-dependently prevented excitotoxicity-induced degranulation of brain MCs and decreased the number of MCs (162). However, it is unknown whether TRESK channels are expressed in MCs, and the MC stabilizing effect of cloxyquin needs to be further studied.

#### 3.1.4 Targeting receptors on the MC surface

##### 3.1.4.1 MRGPRX2 antagonists

MRGPRX2 is a member of the Mas-related gene receptor family and it is emerging as a prominent receptor involved in non-IgE-mediated allergic reactions, including urticaria, rosacea, itch, atopic dermatitis and adverse drug reactions (30). Due to its low selectivity and affinity, MRGPRX2 can interact with a diverse group of ligands such as neuropeptides, antimicrobial peptides and FDA-approved drugs (72), playing a critical role in promoting MC-mediated host defence. Naturally occurring compounds such as isoliquiritigenin, shikonin, imperatorin and roxithromysin were shown to bind to MRGPRX2 in molecular docking studies and surface plasmon resonance (33). They could inhibit C48/80 or substance P (SP)-induced passive cutaneous anaphylaxis in mice. Although a series of natural and small-molecule MRGPRX2 antagonists were discovered, none of them is in clinical use. Combining new protein analysis techniques such as cryoEM and X-ray crystallography with three-dimensional (3D) structure analysis of MRGPRX2 antagonist complexes will enable the rational design of MRGPRX2 antagonists with higher affinity.

##### 3.1.4.2 KIT antagonists

KIT inhibitors can inhibit the binding of stem cell factor (SCF) to the KIT receptor thereby inhibit KIT-dependent MC activation. These agents can be classified on the basis of their parent scaffolds into six categories, including pyrimidine derivatives, particularly N-phenyl-2-pyrimidine-amine, quinazoline derivatives, indolinone derivatives, particularly pyrrol-substituted indolinones, indole derivatives, quinoline derivatives and others (73–77). KIT inhibitors are usually known as anti-cancer agents. They also have therapeutic value for the treatment of MC-related diseases, especially chronic allergic asthma and mastocytosis. The long-term treatment with KIT antagonists such as avapritinib can cause a sustained decrease in serum tryptase levels and the virtual eradication of tissue MCs (78). However, the use of KIT inhibitors must be balanced against their potential side effects. Although KIT inhibitors have the advantages of immediate, complete, sustained and non-toxic remission in anti-allergy, this new drug indication warrants further studies in patients with allergic diseases (74–76).

##### 3.1.4.3 NK-1 receptor antagonists

NK-1 receptor is generally localized to skin MCs and considered to play a vital role in stress-induced inflammation when it combines with the substance P (79). CP99994, (2S,3S)-3-(2-methoxybenzylamino)-2-phenylpiperidine (**Figure 2**) (80), is a representative drug that blocks the binding of substance P to the NK-1 receptor and inhibits NK-1-dependent MC degranulation. As an NK antagonist, CP99994 holds therapeutic potential in the treatment of stress-induced inflammatory skin diseases. No evidence showed that CP99994 also has an inhibitory effect on other activation pathways (79).

##### 3.1.4.4 Silencing MC through inhibitory receptors

Inhibitory surface receptors such as Siglec-6, Siglec-8, FcγRIIB, CD200R and CD300a are able to inhibit MC activity (81–83). Inhibition is mainly accomplished through immunoreceptor tyrosine-based inhibitory motifs (ITIMs) in their cytoplasmic tail that can reverse one or more tyrosine phosphorylation steps critical to progressive signal transduction. The anti-Siglec-8 mAb (lirentelimab) can selectively inhibit MCs and deplete eosinophils. In omalizumab-naive and omalizumab-refractory patients with CSU, lirentelimab decreased the degree of disease activity by 77% and 45% respectively at week 22 according to urticaria activity score (NCT03436797) (84). As the Siglec-8 receptor is also expressed on eosinophils, lirentelimab can reduce the numbers of both blood eosinophil and gastrointestinal MCs in patients with eosinophilic gastrointestinal diseases. Apart from lirentelimab, the CD200R agonist LY3454738 was also developed for the treatment of atopic dermatitis and CSU (85). Bispecific antibodies, that cross-link activated receptors, FcϵRI or KIT, with inhibitory receptors (CD300a or FcγRIIb), could abrogate FcϵRI- or KIT-induced signalling (86, 87). For example, the DARPin-Fc fusion protein can aggregate FcϵRI-bound IgE with FcγRIIb and block IgE-FcϵRI binding faster, indicating that it is more efficient than omalizumab, causing the dissociation of preformed ligand-receptor complexes (85).

### 3.2 Interfering intracellular signalling pathways

MC activation is accomplished by various signalling enzymes. Inhibiting the activity of these vital enzymes can disturb the signalling pathways and stabilize MCs to some extent.

#### 3.2.1 Src family kinase inhibitors

The SFKs Lyn, Fyn, Bruton’s tyrosine kinase (BTK) and Hck participate at the start of activation-induced MC signalling (88). Suppressing the activity of SFKs can reduce the symptoms of allergic diseases especially asthma and rhinitis (89). A total of >20 different drugs were proved to inhibit SFKs, including synthetic drugs such as saracatinib, dasatinib, ibrutinib and acalabrutinib, and natural drugs such as sophorae flos (**Figure 2**) (90). BTK inhibitors can broadly inhibit FcϵRI-dependent MC activation and cytokine production, thus preventing allergen-induced contraction of isolated human bronchi (91). Acalabrutinib, a BTKi, can completely prevent moderate IgE-mediated anaphylaxis in mice and protect against death during severe anaphylaxis (92). Apart from MC activation, SFKs also play crucial roles in signal transduction and regulation of other cell biological processes, such as proliferation, differentiation and apoptosis; research is focused on the role and mechanism of action of SFKs in tumorigenesis instead of allergic diseases (93).

#### 3.2.2 Syk inhibitors

Syk is a 72 kDa non-receptor tyrosine kinase containing two SRC homology 2 domains and a kinase domain. Its expression is highest in haematopoietic cells (94). Syk acts as a central initiator in the MC activation signal pathway. Inhibitors of Syk can be classified on the basis of their parent scaffolds, represented by pyrimidines, prazolyl, 1,6-naphthyridones, pyrido[3,4-b]pyrazine and their derivatives (95–98). These compounds inhibit Syk kinase by competing with ATP-binding sites (99, 100). The most widely studied Syk inhibitors in clinical trials are R112, R406, R788 and R343 (94, 101) which belong to the 2,4-diaminopyrimidine family. Except for small molecule compounds, natural substances such as piceatannol and curcumin also possess inhibitory activity of Syk (102, 103). A phase II study showed that intranasal dosing of Syk inhibitors showed a rapid onset of action without serious side effects in the treatment of allergic rhinitis (94). However, as in the case of SFK inhibitors, Syk is also a vital signal transducer of activated immunoreceptors in multiple downstream events, which differ depending on the cell type, including proliferation, differentiation and phagocytosis. Therefore, except for allergic diseases, Syk inhibitors were also reported to be an attractive target for therapeutic interventions for autoimmune and inflammatory diseases, cancer and infectious diseases, including rheumatoid arthritis, leukemias and plasmodium falciparum malaria (104).

#### 3.2.3 PKC family inhibitors

PKC is a family of protein kinase enzymes that phosphorylate hydroxyl groups on threonine and serine amino acid residues to control the protein function in MC activation pathways (105). The PKC structure consists of a catalytic C-terminal domain and a regulatory N-terminal held together by a hinge region (106). Based on their different sites of action, PKC inhibitors can be classified into catalytic and regulatory domain inhibitors. Clinical trials of PKC candidates are mainly focused on those that inhibit the catalytic domain. The catalytic domain is a highly conserved region throughout the PKC family, making it challenging to selectively target a particular isoform (107). The optimal structure of the catalytic domain inhibitor is bisindolylmaleimide, a staurosporine analog, while the two optimal inhibitors of the regulatory domain are calphostin C and sphingosine (**Figure 2**) (106, 107). Apart from synthetic small-molecule inhibitors, a couple of natural compounds also have inhibitory activity, including quercetin and myricitrin (108, 109). PKC inhibitors can be used in systemic mastocytosis and asthma (110). Skin and lung MCs were shown to be more sensitive to PKC downregulation than other MCs (111), indicating a good therapeutic effect of PKC inhibitors for patients with skin and airway allergic diseases. Except for the MC activation pathway, PKCs are also involved in multiple signal transduction systems that control cell proliferation, differentiation, apoptosis, survival, migration and invasion. Studies recommend that PKC inhibitors can be applied to other diseases including cancer, neurological and cardiovascular diseases, and infections (112).

#### 3.2.4 PI3K inhibitors

PI3Ks are a family of intracellular heterodimeric lipid kinases responding to environmental factors such as nutrition and growth factors. They regulate a variety of biological functions, including cell growth, differentiation, proliferation, metabolism, genomic stability, motility, angiogenesis and protein synthesis (113). Idelalisib (**Figure 2**) is a potent and representative PI3K inhibitor that was approved for the treatment of non-Hodgkin lymphoma and chronic lymphocytic leukemia (114–116). It binds noncovalently and reversibly to PI3K p110d in its ATP-binding site (117). To assess its therapeutic effect in allergic disease, 41 patients with allergic rhinitis received idelalisib (100 mg twice daily) or a placebo for 7 days, and then received an allergen challenge on day 7 (NCT00836914) (118). After a 2-week washout period, subjects received the alternate treatment, and the allergen challenge was repeated. The study demonstrated that idelalisib reduced the allergic response after the allergen challenge with notable therapeutic effects regarding nasal symptoms, airflow and secretion weight compared with the placebo. No marked side effects were observed in patients at a dose of 100 mg idelalisib twice daily over the period of 7 days (118).

#### 3.2.5 Other enzyme inhibitors

Apart from the aforementioned enzymes, a number of other enzymes involved in MC signalling pathways can also be the target of MC stabilizers: i) Janus kinase 3 (JAK3) is a member of the JAK family of tyrosine kinases and is involved in cytokine receptor-mediated intracellular signal transduction. WHI-131 is the inhibitor of JAK3 which inhibits both Ca^2+^ ionophore and IgE-mediated MC degranulation (119); ii) Ceramide Kinase (CERK) acts as a Ca^2+^-sensor for MC activation. K1 is the inhibitor of CERK which can notably suppress both Ca^2+^ ionophore and IgE-mediated MC activation (120). K1 does not inhibit IgE-/antigen-induced tyrosine phosphorylation or subsequent Ca^2+^ increase, indicating a distinctive pathway (121); iii) PDEs catalyse the hydrolysis of 3’,5’-cyclic adenosine monophosphate (cAMP) and 3’,5’-cyclic guanosine monophosphate (cGMP) in cells. Both cAMP and cGMP are important secondary messengers involved in MC activation. As an inhibitor of PDE5, vardenafil was found to ameliorate MC-mediated allergic reactions and reduce histamine release (122), providing evidence for the potential MC-stabilizing properties of PDE inhibitors.

#### 3.2.6 Nanoenzyme

Recently, ceria nanoparticles (CeNPs) were reported to be an effective phosphatase-mimetic MC nano-stabilizer protecting against allergic diseases (123). The regenerable catalytic hotspots of surface oxygen vacancies endow CeNPs with sustainable and excellent phosphatase-mimetic activity. The CeNPs can block the phospho-signalling cascades of MC activation, and thus inhibit the degranulation of allergic mediators and the resulting pathological responses (123). Compared with natural enzymes, the nanoenzyme shows advantages such as low-cost, easy preparation and high stability under harsh conditions (124). Its structure can further be modified to improve its targeting, providing a new research foundation for the development of MC stabilizers.

### 3.3 MC stabilizers disturbing MC degranulation

#### 3.3.1 Hybrid proteins

MC exocytosis is accomplished by a number of proteins, such as SNAP 25, synapbrevin, syntaxine and others (125). If one of these proteins is inactivated, for example via protease cleavage, the degranulation machine will be broken. A hybrid protein that consists of at least one MC-targeted protein and one protease was reported to disturb MC exocytosis. The protein must attach to the MC or be taken up by the MC, including IgE or anti-IgE. The protease cleaves one or several proteins involved in MC secretion, which includes botulinum or tetanus toxin light chains and Neisseria gonorrhoea IgA protease. This hybrid protein can be synthesized *in vitro* or in the appropriate host cells by the gene recombination technology (fusion of protease and light chain genes). To date, these hybrid proteins have not entered clinical application. From animal studies, the recommended uses for allergic diseases include asthma, allergic dermatitis and allergic desensitization. It can also be taken as a preventive treatment when taking drugs with fatal allergic side effects (125).

#### 3.3.2 Statins

Statins are a class of 3-hydroxy-3-methylglutaryl coenzyme A (HMG-CoA) reductase inhibitors commonly used to treat hypercholesterolemia, including fluvastatin, simvastatin and atorvastatin. They bind to low-density lipoprotein (LDL) receptors expressed on MCs and suppress geranylgeranyl transferase by depletion of intracellular mevalonic acid, which deactivates small GTP-binding proteins (126). GTP-binding proteins are involved in microtubule formation. Owing to the importance of microtubules in granule translocation, granule secretion will be inhibited.

A number of studies have identified the therapeutic values of statins for the treatment of asthma. Fluvastatin was reported to suppress peripheral blood mononuclear cell proliferation and inflammatory responses in patients with allergic asthma (127). Simvastatin was shown to inhibit airway hyper-responsiveness in a murine model (128). The oral atorvastatin 40 mg daily, in conjunction with inhaled corticosteroids, was shown to improve lung function and reduce sputum macrophage counts in patients with mild-to-moderate atopic asthma (NCT00126048) (129). Furthermore, statins were demonstrated to have potential therapeutic value in the treatment of MC-related skin diseases, including alopecia areata, atopic dermatitis, psoriasis and mastocytosis (130). Fluvastatin was the most potent MC activation inhibitor among statins, suppressing IgE-induced cytokine secretion (131). Although statins may be useful for the treatment of MC-related diseases, they are mainly used to treat hypercholesterolemia.

### 3.4 MC stabilizers with mixed action mechanism

#### 3.4.1 Flavonoids

Flavonoids are naturally occurring compounds with potent anti-inflammatory, antioxidant and MC-blocking activities, including fisetin, genistein, quercetin, luteolin and kaempherol. The backbone of flavonoids is similar to a part of the structure of cromolyn, and numerous flavonoids were identified possessing MC stabilization activity. Fisetin was proved to interfere cell-to-cell interaction between MC and T cell membranes, and inhibits the activity of NF-κB and MAPKs (132). Genistein inhibits proinflammatory cytokine production of human MCs through the suppression of the ERK pathway (133). Quercetin can decrease the activity of the PKC family. Compared with cromolyn, it is more effective in blocking MC cytokine release and treating contact dermatitis and photosensitivity (134). Luteolin (lut) and its novel structural analog 3’,4’,5,7-tetramethoxyluteolin (methlut) were also proposed as more effective MC stabilizers than cromolyn regardless of the trigger and the mediator measured (135). Methlut was more effective in inhibiting β-hexosaminidase (β-hex), TNF and histamine secretion. The mechanism of action for methlut may be due to its ability to inhibit intracellular Ca^2+^ increase, as well as NF-κB induction at both the transcriptional and translational levels without affecting cell viability.

Flavonoids were reported to be effective in the treatment of allergic conjunctivitis, rhinitis, otitis, asthma and food allergy. A patent demonstrated that flavonoids can also be used together with proteoglycans including chondroitin, keratan and dermatan sulfates to treat MC activation-induced diseases (136).However, the hypothesis has not been verified *in vivo.* Apart from flavonoids, a number of other naturally occurring compounds were reported to stabilize MCs including thymoquinone, capsaicin, coumarins, phenols, terpenoids and amino acids (137–142). They were proved to inhibit MC degranulation and decrease the number of MCs, such as thymoquinone and capsaicin (141, 142). Most of them are complex structures, and the precise mechanism by which they act remains largely unknown. It is hypothesised that, as in the case of flavonoids, a number of segments of the allergic signal cascade are targeted.

#### 3.4.2 Pemirolast

Pemirolast or (9-Methyl-3-(1H-tetrazol-5-yl)-pyrido[1,2-a]pyrimidin-4-one; **Figure 2**), is a [1,2-a]pyrimidin-4-one derivative with MC stabilizing properties. Novel compounds derived from pemirolast have been found according to the deuterium kinetic isotope effect. The structural formula is shown in **Figure 2**. R1-R8 can be hydrogen- or deuterium-independent with at least one deuterium. Deuterium-enriched compound would not cause any additional toxicity since D_2_O or DHO is formed during drug metabolism. Other elements may also be selected from less prevalent isotopes including ^13^C or ^14^C for carbon, ^33^S, ^34^S, or ^36^S for sulphur, ^15^N for nitrogen, and ^17^O or ^18^O for oxygen. Pemirolast and its derivatives can be potent inhibitors of Ca^2+^ uptake and release from intracellular stores. They can also suppress phosphodiesterase activity, increase intracellular cAMP levels, and inhibit arachidonic acid release and metabolism (143).

Pemirolast can inhibit both antigen and CP48/80-induced MC degranulation (144). It can be used in allergic diseases, especially allergic asthma and ragweed allergic conjunctivitis. In the treatment of ragweed allergic conjunctivitis, pemirolast potassium 0.1% is as efficacious and safe as nedocromil sodium 2% but is superior in comfort during topical application (145). Thus, pemirolast may be more suitable for continuous therapy (145). To obtain a better curative effect, pemirolast can be combined with other agents used in the treatment of MC degranulation-mediated diseases.

#### 3.4.3 Tacrolimus and cyclosporine

Tacrolimus and cyclosporine (**Figure 2**) are immunomodulatory agents usually used to treat autoimmune diseases. Both cyclosporine (5mM) and tacrolimus (5mM) were shown to inhibit cytokine release from MCs. Cyclosporine effectively inhibits Ca^2+^-dependent protein phosphatase activity in MCs, while tacrolimus is considerably less effective (146). However, tacrolimus is ~100 times more potent than cyclosporine as an inhibitor of IgE-dependent MC degranulation, meaning that tacrolimus can stabilize MCs in a variety of ways (146). Although tacrolimus can stabilize MCs, it is not typically used in the treatment of MC related diseases (146).

#### 3.4.4 Artesunate

Artesunate (**Figure 2**) is usually used in the treatment of malaria. Besides, artesunate was found to inhibit Syk and PLCγ1 phosphorylation, IP_3_ formation, intracellular Ca^2+^ increase in MCs (147) as well as downregulate T helper 17 cell responses (148). It can block IgE-mediated MC degranulation in a dose-dependent manner (147). In animal models, artesunate has a protective effect in allergic asthma and showed anti-inflammatory effects similar with dexamethasone (149).

#### 3.4.5 Endogenous stabilizers of MCs

Endogenous stabilizers of MCs play a vital role in controlling activation of MCs and consequently in the immune homeostasis of the body. For instance, as an endogenous cannabinoid, anandamide inhibited the degranulation of dural MCs through CB2 receptors on the surface of MCs (163). Heparin, chondroitin sulphate and spermine from MCs can inhibit the activation of MCs (164). Additionally, there are different endogenous molecules which can inhibit MC activation such as progesterone, testosterone, corticosterone and 2-arachidonoyl glycerol (2-AG) (164).

## 4 Discussion

The widespread tissue distribution and versatility of MCs make them a hotspot in the studies of related diseases/disorders. They exert their function mainly through mediators released during activation. Inhibiting one of the activated receptors on the MC surface will prevent MCs from being activated by certain substances. Apart from inhibiting activated receptors, activating inhibitory receptors on MCs can also stabilize them. Co-aggregating inhibitory receptors with activated receptors can reverse the activation mediated by the latter, providing a new direction for research and development of MC stabilizers.

The influx of extracellular Ca^2+^ is a vital event of MC degranulation. Inhibiting Ca^2+^ influx is the mechanism of action of most clinical MC stabilizers, represented by cromolyn sodium. Besides, interfering Cl^-^ and K^+^channels can also influence the Ca^2+^ influx indirectly through their effects on cell membrane potential, which may offer a novel method for the treatment of MC-related diseases/disorders. Together with the influx of extracellular Ca^2+^, a series of phosphorylation cascades contribute to the activation of MC. Inhibiting the enzymes involved in MC activation signal pathway can prevent MC degranulation. However, these enzymes are not usually specific to MC activation pathway. They always participate in other cell biological processes, such as proliferation, differentiation and apoptosis. This may explain why enzyme inhibitors without MC-targeting are often not used as MC stabilizers in clinical practice, and clinical drugs for other indications including stains, tacrolimus, cyclosporine and others may have a MC stabilization activity. Low targeting is an urgent problem for MC stabilizers. The mAbs and humanized sdabs are the epoch-making progress of the development of MC stabilizers with high targeting and minimal side effects. There are three generations of anti-IgE mAbs including omalizumab, ligelizumab, quilizumab and UB-221. The improvement of each generation is mainly focused on the affinity for IgE and the reduction of IgE production. Compared with mAbs, sdabs exhibit high production yield in simple expression systems and extraordinary stability. State-of-the-art strategies for multiple targeting and half-life extension can be easily applied to them.

Progress in new material therapy technology has promoted the development of MC stabilizers. Normally, nanomaterials used for medical applications are synthesized using specific polymers, lipids, nano enzymes or proteins. Cell surface receptors, such as FcϵRI or MRGPRX2, can be applied to target nanoparticles (NPs) to specific subsets of MCs. By recognizing the receptors on MCs in their tissue environments, NPs could be tailored to alleviate symptoms such as specific allergies in individual patients, thereby opening a new frontier in precision therapeutics. Furthermore, with the increasing interest in genetic modification, nucleic acid-containing NPs could be engineered to modify the gene necessary for regulating MC activation. MC activation does not always lead to pathology. A recent study showed that MC degranulation suppresses epileptic seizures through the serotonin in their granules (165). Thus, the approach based on genetic modification may interfere with MC-mediated inflammatory responses while preserving protective innate immune or tissue homeostatic functions (166). At present, the development of nano MC stabilizers is still at an early stage and has a broad scope in field of allergy research in the future.

MC leukemia (MCL) is a rare form of systemic mastocytosis with poor prognosis (167). Patients with MCL may benefit from MC-targeted therapies, including controlling MC-related symptoms and cytoreductive therapy. Interferon-α and steroids are usually used to control clinical symptoms. However, their effect is transient and limited (167). With the discovery of KIT mutations in MCL, KIT antagonist have become a hot spot in MCL therapy (168). KIT antagonist represented by midostaurin has been approved for patients with MCL (169) due to its strong inhibitory activity on neoplastic human MC carrying the KIT D816V mutation in preclinical and clinical settings (167). Avapritinib, dasatinib, masitinib and imatinib also hold therapeutic effects in MCL with different KIT mutated forms (167). Data detailing mutations are useful in supporting individualized treatment. Despite achieving initial success, the efficacy of these KIT-targeting agents on MCL prognosis requires further evaluation. Cytostatic drugs (represented by 2-CdA) have been shown to induce apoptosis of the human MC (169). However, these drugs may have unpredictable toxicity and adverse effects. Whether patients with MCL may benefit from combination polychemotherapy remains unknown. Chemotherapy combined with targeted therapy might be an interesting direction for MCL treatment.

Future studies of MC stabilizers should focus on the following: i) Using targeted agents or new material technology to develop more effective and targeted MC stabilizers; ii) preventing the detrimental response caused by MC activation while preserving its vital roles in host defence; iii) exploring and developing novel humanized MC culture technology and construction of humanized animal models to investigate the application of MC stabilizers; iv) increasing the number of clinical trials; and v) isolating compounds from biological agents as a promising way to develop new MC stabilizers.

## Author contributions

MC: Writing – original draft, Writing – review & editing. YG: Writing – review & editing.
